# A common *SSD1* truncation is toxic to cells entering quiescence and promotes sporulation.

**DOI:** 10.17912/micropub.biology.000671

**Published:** 2022-12-09

**Authors:** Linda Breeden, Shawna Miles

**Affiliations:** 1 Fred Hutchinson Cancer Center, Basic Science Division, Seattle, WA, USA

## Abstract

Ssd1p is an RNA binding protein in *Saccharomyces cerevisiae* that plays an important role in cell division, cell fate decisions, stress response and virulence. Lab strain W303 encodes the terminal truncation *ssd1-2,* which is typically interpreted to be a loss of function allele. We have shown that *ssd1-2* is toxic to *mpt5-Δ* mutants and to diploids entering stationary phase and quiescence. The *ssd1-Δ* null shows no toxicity, indicating that *ssd1-2* is disrupting an essential function that does not solely require Ssd1p. *ssd1-2* cells are also more sensitive to stress than *ssd1-Δ* . These phenotypes are recessive to *SSD1-1* . In contrast, *ssd1-2* plays a dominant role in promoting sporulation.

**Figure 1. The ssd1-2 truncation found in W303 is toxic to cells entering quiescence but promotes sporulation.**
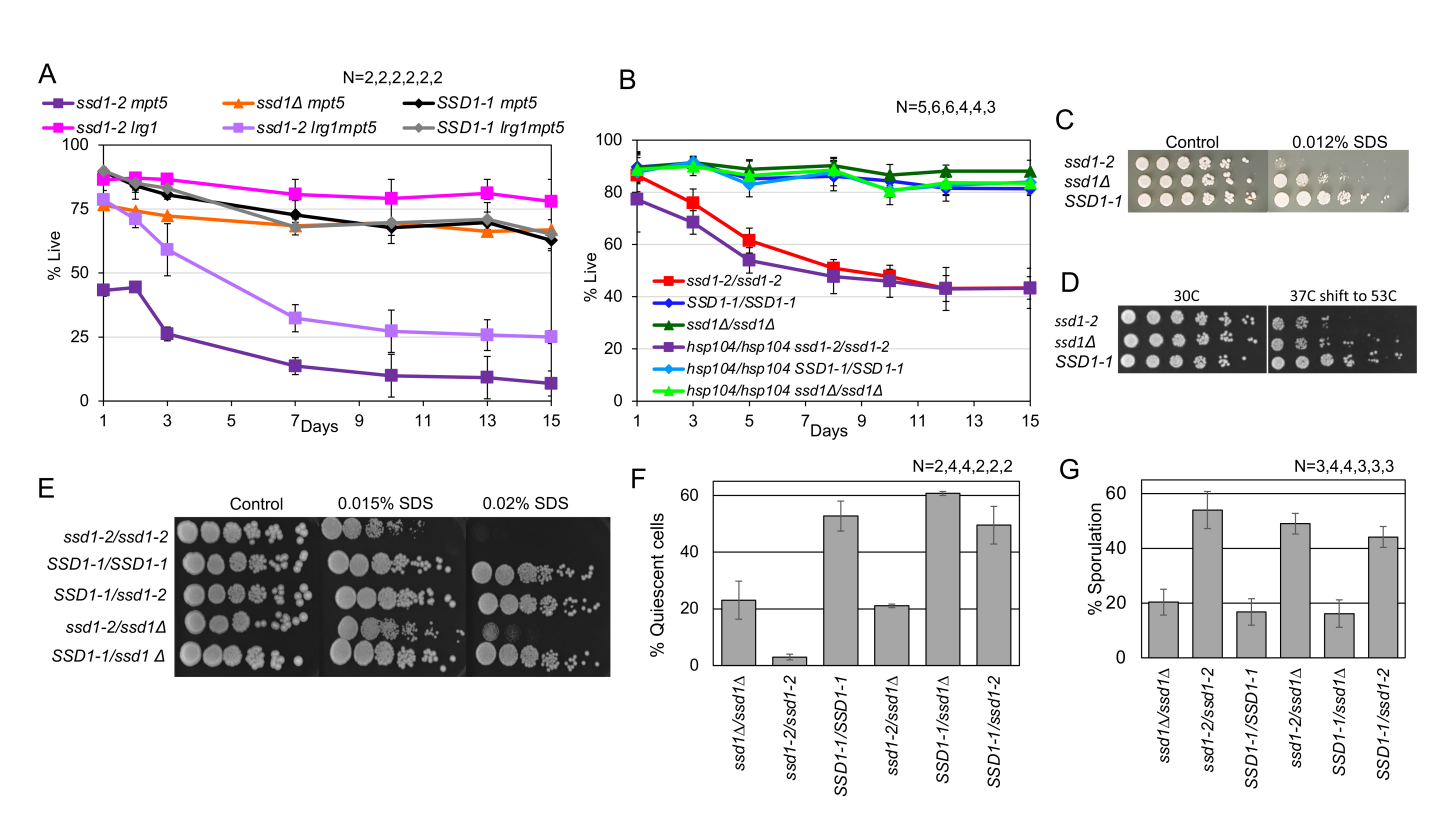
The genotypes used in each plot are indicated in the legend and the number of replicates is listed above, in legend order. (A and B) Percent of viable cells as a function of days after inoculation into rich glucose medium. (C) Innate tolerance to growth on plates containing the detergent Sodium Dodecyl Sulfate (SDS). (D) Acquired stress tolerance induced by treating cells for 30 minutes at 37° C, then shifting to 53° C for 30 minutes before making serial dilutions and plating. (E) Increased sensitivity of *ssd1-2* to SDS is recessive. Innate tolerance to SDS in diploids, genotypes indicated on the left. (F) Quiescence defect of *ssd1-2* is recessive. Percent of dense quiescent cells purified from seven day cultures. (G) Sporulation promoted by *ssd1-2* is dominant. Percent of spore-containing asci induced by limiting cells for nitrogen and glucose.

## Description

Ssd1p is a highly conserved pseudonuclease in the Dis3/RNAse II family. Active site mutations have arisen multiple times but there has been broad conservation of other regions, indicating that it has evolved critical functions other than RNA cleavage (Ballou *et al.* 2021). Ssd1p has retained its RNA binding capacity and its 300 associated RNAs are highly enriched for cell wall and cell cycle transcripts (Hogan *et al.* 2008; Jansen *et al.* 2009; Hose *et al.* 2020). Full length *SSD1* rescues the v iability of *sit4, swi4* and many other mutants, and these mutants d ie in the presence of the truncated allele (Breeden and Nasmyth 1987; Ogas *et al.* 1991; Sutton *et al.* 1991). Hence, the unconventional name *SSD1-V* was given to the full-length allele found in S288c, and *ssd1-d2* was given to the truncated allele found in the common lab strain W303 *.* We refer to the S288c allele as *SSD1-1* and the W303 allele as *ssd1-2* (Miles *et al.* 2019). In most assays, the truncation *ssd1-2* has been interpreted to have a null phenotype. Terminal truncations of Ssd1p have arisen independently seven times in one thousand sequenced *S. cerevisiae* strains, and the *ssd1-2* truncation is the most common, as it arose in four of the seven strains (Peter *et al.* 2018).

We found that SSD1-1p and another RNA binding protein encoded by *MPT5* have parallel roles in promoting the survival of W303 haploids as glucose becomes limiting and one or the other is required for the successful transition to quiescence (Li *et al.* 2013; Miles *et al.* 2019). We also found that Ssd1-1p promotes survival and quiescence in W303 diploids. *ssd1-2ssd1-2* diploids cannot enter quiescence but they can sporulate, and introducing *SSD1-1* is sufficient to enable quiescence entry and disrupt sporulation. As with the *ssd1-2mpt5-Δ* haploid, the *ssd1-2/ssd1-2* diploid loses viability rapidly as glucose becomes limiting and survival can be rescued by the addition of trehalose (Miles *et al.* 2019). Trehalose serves as a protectant from oxidation, desiccation and other forms of cell wall stress (Elbein *et al.* 2003). Cell wall fortification is a key step in the formation of quiescent cells (Li *et al.* 2015), and eight of the cell wall mRNAs bound by Ssd1p are present at up to five-fold higher levels in haploids than in diploids (de Godoy *et al.* 2008). Three of these genes are haplo-insufficient (Pir *et al.* 2012). These observations indicate that diploids have a limited supply of cell wall proteins, so our working hypothesis was that loss of Ssd1p function in protecting critical cell wall mRNAs may be lethal in diploids. In the smaller haploids, Mpt5p may compensate for its loss.

However, further study has shown that this is not the explanation. When we deleted Ssd1p, we expected to see the same lethality we observed with *ssd1-2mpt5-Δ* and *ssd1-2/ssd1-2* diploids as they entered stationary phase, but that was not the case (Figure 1 A and B.) Eliminating *SSD1* ( *ssd1-Δ* ) rescued the viability of both strains (after 15 days, *ssd1-Δmpt5-Δ* versus *ssd1-2mpt5-Δ* p=0.0033, *ssd1Δ/ssd1Δ* versus *ssd1-2/ssd1-2* p=10 ^-7^ ). We conclude that ssd1-2p persists in an altered form that is toxic to cells as they transition from a proliferative to a non-proliferative state. This led us to ask if *ssd1-2* has a more extreme phenotype in other contexts. Figure 1C shows that *ssd1-2* is innately more sensitive to detergent than *ssd1-Δ* . *ssd1-2* is also more sensitive than *ssd1-Δ* to temperature stress. Cells can acquire thermo-tolerance if they are first incubated at 37° C to induce the heat shock response and then shifted to 53° C for 30 minutes. *ssd1-2* is less thermo-tolerant than *ssd1-Δ* in this assay (Figure 1D). The innate stress tolerance phenotype of *ssd1-2* is recessive (Figure 1E), indicating that it cannot override the *SSD1-1* allele in this assay, but it is toxic on its own.

Surviving to quiescence (Breeden and Tsukiyama, 2022) and recovering from cell wall damage both involve activation of the C ell W all I ntegrity pathway (Torres *et al.* 2002; Quilis *et al.* 2021). This pathway is constitutively activated in *ssd1-Δ* (Arias *et al.* 2011), but not in the *ssd1-2* strain (Stewart *et al.* 2007). CWI is also not activated in *ssd1-2mpt5-Δ* , because one of Mpt5p’s primary targets of inhibition is Lrg1p, which is a negative regulator of the CWI pathway (Stewart *et al.* 2007). To see if failure to activate CWI is responsible for the loss of viability during stationary phase, we deleted *LRG1* from *ssd1-2mpt5-Δ.* Figure 1A shows that releasing CWI from inhibition by Lrg1p rescues the viability of *ssd1-2mpt5Δ* at day 1, but they lose viability rapidly thereafter (P=0.0073 on day 1, P=0.043 after 15 days).

Ssd1p is also required for the Hsp104p-dependent disaggregation of proteins (Mir *et al.* 2009). This role in relieving proteostatic stress is important for longevity (Moreno and Aldea 2020) and aneuploidy tolerance (Hose *et al.* 2020), which is fairly common in *S. cerevisiae* (Peter *et al.* 2018). Hsp104p also functions in the disaggregation and propagation of prions (Chernoff *et al.* 1995). *ssd1-Δ* prevents Hsp104p-dependent disaggregation but *ssd1-2* retains some activity (Mir *et al.* 2009). We wondered if the truncated *ssd1-2* allele might deregulate, delocalize or otherwise modify Hsp104p disaggregation function and so result in lethality. Figure 1B shows that deleting *HSP104* has no discernible impact on the transition to stationary phase, nor does it rescue the lethality of *ssd1-2* .

Diploids survive periods of nutrient limitation either by entering quiescence or by sporulating and there are wild strains that take one or the other path exclusively (Miles *et al.* 2019). Ssd1p also plays a determining role in this decision. *ssd1-2/ssd1-2* produces one-tenth as many dense quiescent cells as *SSD1-1/SSD1-1* (p=10 ^-4^ ) and one-fifth as many as *ssd1-Δ/ssd1-Δ* (p=0.0026) *,* so it is more deleterious than the null mutant (Figure 1F). The *SSD1-1/ssd1-2* quiescent cell yield is equivalent to *SSD1-1/SSD1-1* (P=0.5462), so *ssd1-2* is recessive. In contrast, the sporulation promoted by *ssd1-2* is dominant (Figure 1G). *SSD1-1/ssd1-2* sporulates at a much higher rate than *SSD1-1/SSD1-1* (p=.0005) and approaches that of *ssd1-2/ssd1-2* (p=.08). This promotion of sporulation is clearly a property of *ssd1-2* , because *ssd1-Δ/ssd1-Δ* behaves just like *SSD1-1/SSD1-1* (p=.37).

Many things, including the ability to manage stress, tolerate aneuploidy, evade host defenses, enter quiescence and sporulate are influenced by the *SSD1* locus. These are responses to common environmental changes that affect survival. Polymorphisms in *SSD1* , particularly the *ssd1-2* truncation, significantly change the cellular response in many contexts, but the properties of this allele have not been systematically studied, because it has been interpreted to result in loss of Ssd1p function. Our data indicate that the *ssd1-2* truncation is not a simple loss of function allele in several key assays. *ssd1-2* is more detrimental than the *ssd1-Δ* null mutant in stress and in the transition to quiescence, and more beneficial than the null or the full-length Ssd1-1p for sporulation. *ssd1-2* toxicity in response to stress is recessive, but its promotion of sporulation is dominant and could explain why this specific Ssd1p truncation arises in natural populations, especially those whose environments favor sporulation over quiescence. It would be of great interest to know what is unique about this ssd1-2p truncation and how it influences these and other cell fates.

## Methods

*Yeast Strains and Growth Conditions*

All strains used are isogenic with BY6500, the prototrophic version of W303 (Li et al., 2009). Replacement of *ssd1-2* with *SSD1-1* was described in (Li et al., 2009). *SSD1* and *MPT5* were deleted using pFA6a- *HIS3MX6* and pFA6a- *KanMX* , respectively (Longtine et al. 1998). *HSP104* was deleted using pAG25 and pAG32 (Goldstein and McCusker,1999). *LRG1* was deleted using pAG32 (Goldstein and McCusker,1999).

*Cell Processing*

Viability assays over 15 days of growth and purification of quiescent cells were described in (Miles et al., 2016). Measures of induced sporulation were previously reported in (Miles et al., 2019). Two-tailed Students t-tests were used to determine significant differences therein. The log phase cultures for spotting assays were washed with water and diluted to an optical density (OD) of 1.0 in water, then they were further diluted to 4096 cells per 2μL, using a Z2 Beckman Coulter Counter (Beckman Coulter, Brea, CA). These cultures and four-fold serial dilutions thereof were spotted on YEPD plates (Miles et al., 2019) with SDS added when the plates were poured. The spots were grown for two days at 30°C. The acquired thermo-tolerance was determined by exposing the 1.0 OD cultures to 37°C for 30 minutes, shifting the cells to 53°C for 30 minutes, then ice for 10 minutes. The cells were diluted as stated above, spotted on YEPD plates, and grown for two days at 30°C.

## Reagents

Strains used in this study

**Table:** 

BY6500	*MATa can1-100 rad5 ssd1-2*
BY6563	*MATa can1-100 rad5 ssd1Δ::HIS3*
BY6641	*MATa can1-100 rad5 SSD1-1*
BY6672	*MATa can1-100 rad5 ssd1-2 mpt5Δ::KanMX*
BY6766	*MATa can1-100 rad5 SSD1-1 mpt5Δ::KanMX*
BY6946	*MATa/MATalpha can1-100/can1-100 rad5/rad5 ssd1-2/ssd1-2*
BY6962	*MATa/MATalpha can1-100/can1-100 rad5/rad5 SSD1-1/SSD1-1*
BY6976	*MATa/MATalpha can1-100/can1-100 rad5/rad5 SSD1-1/ssd1-2*
BY7881	*MATa/MATalpha can1-100/can1-100 rad5/rad5 ssd1-2/ssd1Δ::HIS3*
BY7883	*MATa/MATalpha can1-100/can1-100 rad5/rad5 SSD1-1/ssd1Δ::HIS3*
BY7887	*MATa can1-100 rad5 ssd1Δ::HIS3 mpt5Δ::KanMX*
BY7965	*MATa/MATalpha can1-100/can1-100 rad5/rad5 ssd1Δ::HIS3/ssd1Δ::HIS3*
BY8292	*MATa can1-100 rad5 ssd1-2 lrg1Δ::HPHMX4*
BY8295	*MATa can1-100 rad5 ssd1-2 mpt5Δ::KanMX lrg1Δ::HPHMX4*
BY8297	*MATa can1-100 rad5 SSD1-1 mpt5Δ::KanMX lrg1Δ::HPHMX4*
BY8336	*MATa/MATalpha can1-100/can1-100 rad5/rad5 ssd1-2/ssd1-2 hsp104Δ::HPHMX4/hsp104Δ::NATMX4*
BY8338	*MATa/MATalpha can1-100/can1-100 rad5/rad5 SSD1-1/SSD1-1 hsp104Δ::HPHMX4/hsp104Δ::NATMX4*
BY8340	*MATa/MATalpha can1-100/can1-100 rad5/rad5 ssd1Δ::HIS3/ssd1Δ::HIS3 hsp104Δ::HPHMX4/hsp104Δ::NATM*
